# Interaction between Neighborhood Exposome and Genetic Risk in Child Psychotic-like Experiences

**DOI:** 10.21203/rs.3.rs-5830171/v1

**Published:** 2025-02-18

**Authors:** Yinxian Chen, Qingyue Yuan, Lina Dimitrov, Benjamin Risk, Benson Ku, Anke Huels

**Affiliations:** Department of Epidemiology, Rollins School of Public Health, Emory University, Atlanta, GA, USA; Department of Psychiatry and Behavioral Sciences, Emory University School of Medicine, Atlanta, GA, USA; Department of Epidemiology, Rollins School of Public Health, Emory University, Atlanta, GA, USA; Department of Biostatistics and Bioinformatics, Rollins School of Public Health, Emory University, Atlanta, GA, USA; Department of Psychiatry and Behavioral Sciences, Emory University School of Medicine, Atlanta, GA, USA; Department of Epidemiology, Rollins School of Public Health, Emory University, Atlanta, GA, USA; Department of Biostatistics and Bioinformatics, Rollins School of Public Health, Emory University, Atlanta, GA, USA; Gangarosa Department of Environmental Health, Rollins School of Public Health, Emory University, Atlanta, GA, USA

## Abstract

Persistent distressing psychotic-like experiences (PLE) among children may be driven by genetics and neighborhood environmental exposures. However, the gene-environment interaction to persistent distressing PLE is unknown. The study included 6,449 participants from the Adolescent Brain and Cognitive Development Study. Genetic risk was measured by a multi-ancestry schizophrenia polygenic risk score (SCZ-PRS). Multi-dimensional neighborhood-level exposures were used to form a neighborhood exposome (NE) score. SCZ-PRS was not statistically significantly associated with odds of persistent distressing PLE (OR = 1.04, 95% CI: 0.97, 1.13, *P* = 0.280), whereas NE score was (OR = 1.15, 95% CI: 1.05, 1.26, *P* = 0.003). The association between NE score and persistent distressing PLE was statistically significantly attenuated as SCZ-PRS increased (OR for interaction = 0.92, 95% CI: 0.86, 1.00, *P* = 0.039). The findings indicate that persistent distressing PLE may be driven by detrimental neighborhood exposures, particularly among children with low genetic risks.

## Introduction

Psychotic-like experiences (PLEs), also known as subclinical psychotic symptoms or psychotic experiences, encompass unusual or unreal perceptions, thoughts, or beliefs.^1^ PLEs are one of the earliest signs of psychotic disorders and are common in children,^2, 3^ with a prevalence of 17% among those aged 9–12.^4^ Although PLEs do not guarantee a future psychosis diagnosis, those that persist and cause significant distress may be more strongly associated with future psychopathology, including greater functional and cognitive impairments, as well as increased reliance on mental health services.^5, 6^

Both nature (i.e., genetic inheritance) and nurture (i.e., non-genetic exposures) may play a role in the onset of persistent distressing PLE. Schizophrenia polygenic risk scores (SCZ-PRS), representing the individual’s genetic risk of schizophrenia, have been shown to be associated with a higher risk of persistent distressing PLE among children of European ancestry.^7, 8^ Evidence also shows that non-genetic factors, such as household adversity, sociocultural values, and neighborhood environment, can also affect psychosis symptoms.^9–12^ Our previous work reveals that the neighborhood exposome (NE), the totality of exposure to multi-dimensional neighborhood environmental factors,^13^ is associated with an increased likelihood of persistent distressing PLE.^8^ However, it remains unknown whether the nature of persistent distressing PLE can still be explained by the genetic risk of schizophrenia among children from multiple ancestries. Persistent distressing PLE could be precursors of multiple psychiatric disorders, which may have different susceptibility across ancestries.^14, 15^ Moreover, the interaction between genes and NE contributing to persistent distressing PLE is underexplored. Studies on gene-environment interaction in psychosis and other psychiatric disorders produce mixed results, indicating complex etiology.^16, 17^ Investigating the interaction pattern between genetic risk and NE could help elucidate the complex etiology of persistent distressing PLE and identify vulnerable populations, from which the evidence can be used to precisely inform prevention strategies for early manifestations of psychosis and other disorders.

To address the above gaps, we examined the association between genetic risk, NE, and persistent distressing PLE, as well as the gene-environment interaction in a nationwide cohort with children from multiple ancestry backgrounds in the US. We used a multi-ancestral SCZ-PRS as the genetic risk of persistent distressing PLE. We also used a mixture method, weighted quantile sum (WQS) regression, for a holistic assessment of the NE in relation to persistent distressing PLE.

## Results

### Study population

**Figure 1:**
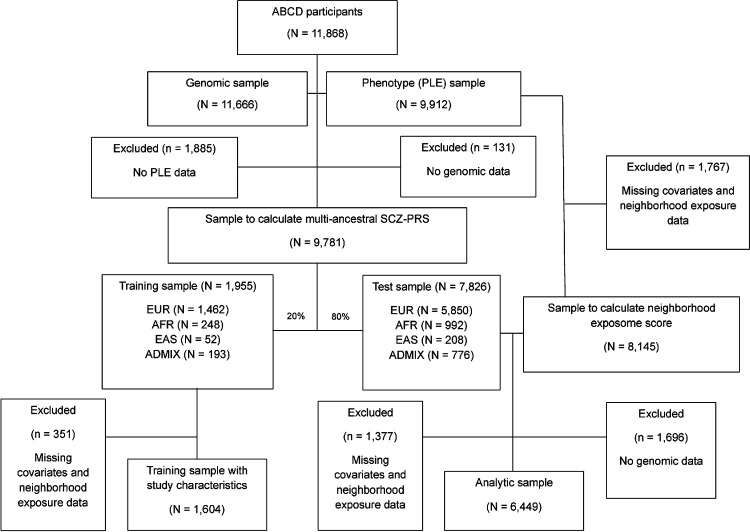
Flowchart of participant selection Abbreviation: ADMIX, Admixed ancestry, AFR, African ancestry; EAS, East Asian ancestry; EUR, European ancestry

This population-based study used data from the Adolescent Brain and Cognitive Development (ABCD) Study. Participants were recruited from 22 US sites between September 2016 and January 2022. Data from baseline and three annual follow-ups were included. The selection process for the analytic sample is shown in Fig. 1 and described in detail in Methods. Briefly, 9,781 participants in the ABCD cohort had both genomic and phenotype (i.e., PLE) data. They were split into training (N = 1,955) and test samples (N = 7,826) for calculating the multi-ancestral PRS using SBayesRC (and PRScsx in sensitivity analysis; see details in Methods). The analytic sample, which is the subset with complete sociodemographic and clinical characteristics of the test sample, included 6,449 participants (Table 1). The mean (SD) baseline age of the participants was 9.9 (0.6) years, and 48% were female. The participants had a mean (SD) income-to-need ratio of 4.4 (3.3), and 66% had at least one parent with a bachelor’s degree or greater. The prevalence of having a family history of psychosis was 8%. Most of the participants reported that their race and ethnicity group was non-Hispanic White (58%), followed by Hispanic (18%) and non-Hispanic Black (11%). Participants were mostly from EUR (77%), followed by AFR (11%) and mixed ancestry (9%). Of all, 1,272 (20%) participants experienced persistent distressing PLE. Due to the random splitting process, the study characteristics were similar between the training sample and the analytic sample (Supplementary Table 1). The analytic sample had more non-Hispanic White participants, higher parental education levels, income-to-need ratio, and more endorsement of persistent distressing PLE compared with participants excluded from the analysis (Supplementary Table 2).

### SCZ-PRS and Persistent Distressing PLE

The distribution of the multi-ancestral SCZ-PRS varied across ancestry groups (Supplementary Fig. 1A). In the total test sample (N = 7,826), SCZ-PRS predicted persistent distressing PLE, with an area under the ROC curve (AUC) of 0.56 (95% CI: 0.55, 0.58) and a Nagelkerke R^2^ of 1.1%. It also predicted persistent distressing PLE in EUR and EAS ancestry groups but did not in AFR and admixed ancestry groups (Supplementary Fig. 1B).

**Figure 2:**
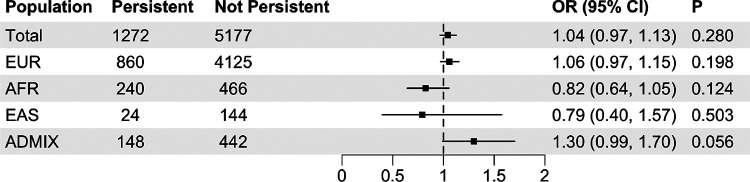
The association between schizophrenia polygenic risk score (calculated by SBayesRC) and persistent distressing psychotic-like experiences Abbreviation: ADMIX, Admixed ancestry, AFR, African ancestry; EAS, East Asian ancestry; EUR, European ancestry; PLE, persistent distressing psychotic-like experiences Note: Models were adjusted for age, sex, income-to-needs ratio (INR), family history of psychosis, parental education levels, and four genetic principal components (PCs)

After adjusting for selected covariates, the SCZ-PRS was not statistically significantly associated with increased odds of persistent distressing PLE (OR = 1.04, 95% CI: 0.97, 1.13, *P* = 0.280) among the total analytic sample (N = 6,449). No statistically significant association between SCZ-PRS and persistent distressing PLE was observed among any of the ancestry groups (Fig. 2).

### NE and Persistent Distressing PLE

**Figure 3:**
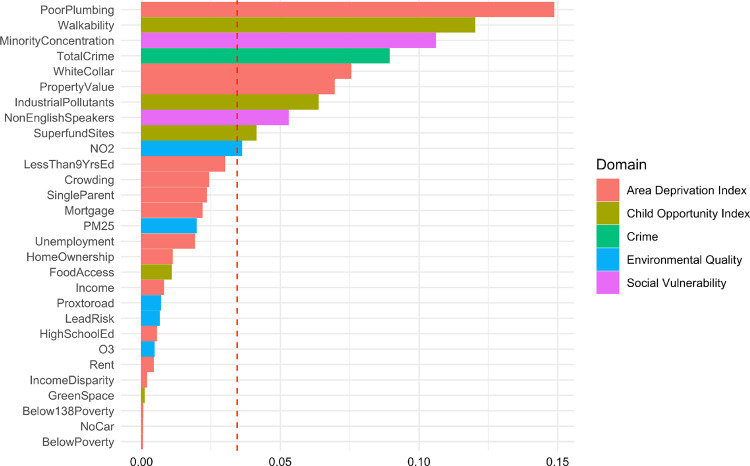
The relative importance of neighborhood factors in neighborhood exposome score estimated by weighted-quantile sum regression Note: The model was adjusted for age, sex, income-to-needs ratio (INR), family history of psychosis, parental education levels, and four genetic principal components (PCs).

Of the 29 neighborhood-level exposure variables in the NE score, the top five exposures with the greatest weight were poor plumbing, lack of walkability, greater minority concentration, greater total crime, and lower percentage of those working in white-collar occupations (Fig. 3). After adjusting for selected covariates, a one-decile increase in NE score was associated with a 15% (OR = 1.15, 95% CI: 1.05, 1.26, *P* = 0.003) increased odds of persistent distressing PLE among the total analytic sample.

### Interaction between SCZ-PRS and NE

**Figure 4:**
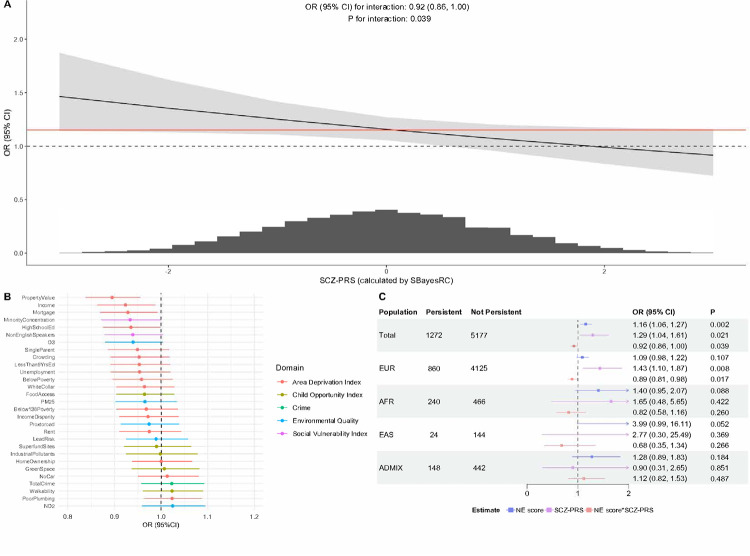
Multiplicative interaction between schizophrenia polygenic risk score (calculated by SBayesRC) and neighborhood exposome score on persistent distressing psychotic-like experiences Abbreviation: ADMIX, Admixed ancestry, AFR, African ancestry; EAS, East Asian ancestry; EUR, European ancestry; NE, neighborhood exposome; PLE, persistent distressing psychotic-like experience; SCZ-PRS, schizophrenia polygenic risk score (A) The association between neighborhood exposome score and persistent distressing psychotic-like experiences across polygenic risk score for schizophrenia; (B) Interaction between polygenic risk score and individual neighborhood factors; (C) Interaction between polygenic risk score and neighborhood exposome score among different ancestries. The interpretation for each term would be (1) OR of persistent distressing PLE for a one-decile increase in NE among those with a population mean SCZ-PRS (NE score); OR of persistent distressing PLE for a one-SD increase in SCZ-PRS among those in the first decile of NE score (SCZ-PRS); OR of the interaction between NE score and SCZ-PRS (NE score*SCZ-PRS). Note: Models were adjusted for age, sex, income-to-needs ratio (INR), family history of psychosis, parental education levels, and four genetic principal components (PCs).

SCZ-PRS modified the association between the NE score and persistent distressing PLE on the multiplicative scale. Among the total analytic sample, the association between NE score and persistent distressing PLE statistically significantly attenuated (OR for interaction = 0.92, 95% CI: 0.86, 1.00, *P* = 0.039) as SCZ-PRS increased (Fig. 4A). The multiplicative interaction between SCZ-PRS and NE score was mainly contributed by neighborhood variables measured by ADI (Fig. 4B). A statistically significant interaction was also observed among the EUR ancestry group (OR for interaction = 0.89, 95% CI: 0.81, 0.98, *P* = 0.017). No evidence of multiplicative interaction was found among other ancestries (Fig. 4C). When following the same approach as the multiplicative interaction, there was no statistically significant additive interaction between SCZ-PRS and the NE score among the total analytic sample (RERI = −0.06, 95% CI, −0.15, 0.03, *P* = 0.189) or any of the individual ancestry groups (Supplementary Fig. 2).

### Sensitivity Analyses

The association between SCZ-PRS calculated by PRScsx and persistent distressing PLE and the interaction with NE score followed the same trend as the primary analysis using SCZ-PRS calculated by SBayesRC, although it was statistically insignificant (Supplementary Fig. 3, 4). Similarly, results from additionally adjusting for self-reported race and ethnicity and excluding AFR ancestry followed the same trend as the primary analysis (Supplementary Table 3, 4).

## Discussion

In this study, we examined the association between genetic risk, NE, and persistent distressing PLE, as well as the interaction between genetic risk and NE, among US children from multiple ancestry backgrounds in the ABCD cohort. Genetic risk was measured by a multi-ancestral SCZ-PRS, and an NE score was calculated using WQS regression incorporating multi-dimensional neighborhood factors. We found that the NE score was statistically significantly associated with increased odds of persistent distressing PLE. A statistically significant interaction was observed between SCZ-PRS and NE score, in which the association between NE score and persistent distressing PLE was attenuated as SCZ-PRS increased.

We found little evidence that the multi-ancestral SCZ-PRS was associated with persistent distressing PLE. The multi-ancestral SCZ-PRS still had acceptable prediction properties in the EUR ancestry group, consistent with previous studies showing the positive association between SCZ-PRS and persistent distressing PLE among the EUR children.^7, 8^ However, although we used the latest developed multi-ancestry PRS approach, SBayesRC, it performed poorly among children from AFR and admixed ancestries, representing 20% of the total analytic sample. Therefore, the noise from those of AFR and mixed ancestries could have prevented us from detecting the genetic risk of persistent distressing PLE when using the multi-ancestral SCZ-PRS. This could be due to several reasons. First, among the GWAS summary statistics of schizophrenia used to train the multi-ancestral SCZ-PRS, those of AFR ancestry had the smallest sample size, inherently encompassing a considerable variance.^18^ However, AFR is the second-largest ancestry group following EUR in the study population, which also means the admixed population comprises a significant proportion of AFR background. The under-representation of multi-ancestral SCZ-PRS for AFR and admixed populations could result in a poor performance in predicting persistent distressing PLE in the study population. Meanwhile, persistent distressing PLE is not exclusively a predictor of schizophrenia but also of broader psychiatric disorders,^19^ of which the genetic risk could vary across ancestries.^14, 15^ The genetic risk of other psychiatric disorders may more accurately represent that of persistent distressing PLE among AFR and admixed populations.

Consistent with our previous work in the ABCD cohort,^20^ we found that NE was associated with increased odds of persistent distressing PLE. Extending the evidence, we found the association is stronger among individuals with a low genetic risk according to the observed negative interaction between NE and SCZ-PRS. The same gene-environment interaction pattern has been observed in twins from the UK and Sweden, in which the variance of psychotic experiences explained by environmental exposures decreased when genetic heritability increased.^21^ In contrast, some previous studies found adverse socio-environmental exposures synergistically interacted with genetic liability in psychotic symptoms and diagnosis of schizophrenia among the European population.^22, 23^ Besides differences in the study population, measures of genetic risk, and types of environmental factors that could contribute to these inconsistent results, the authors of these studies measured environmental factors based on retrospective self-report records as opposed to our objective measures. Individuals with psychotic symptoms may report exposure to adverse environmental factors differentially compared to those without. Recall bias could then arise and affect the validity of interaction estimates. Our findings suggest the predisposition for the development of persistent distressing PLE may vary depending on population characteristics, in which the population with low genetic risk may be more susceptible to neighborhood environmental exposures in developing psychotic outcomes. The reason for the negative interaction remains unclear. A high genetic risk may establish a stable trajectory for persistent distressing PLE that limits the impact of some neighborhood factors (e.g., factors from the ADI domain), resulting in the attenuated association of NE with persistent distressing PLE. However, imprecise additive interaction results caution against interpreting observed statistical interactions as biological effects.^24^

The study has several limitations. First, as mentioned above, the SCZ-PRS performed poorly in AFR and admixed populations in predicting persistent distressing PLE. Although we applied the latest multi-ancestral PRS approach, it did not sufficiently overcome this limitation. Second, participants in the ABCD cohort with incomplete records of all included variables were excluded from the analytic sample. The difference in characteristics between included and excluded participants may indicate a risk of selection bias. Third, PLE was measured by a self-reported questionnaire, which may introduce outcome misclassification. Fourth, we only had data on baseline neighborhood exposures and could not examine the sensitive period in which environmental factors may have a more pronounced effect on long-term psychosis risk. Finally, residual confounding may remain due to unmeasured confounders and misclassification/measurement error of included covariates.

## Conclusion

NE was associated with increased odds of persistent distressing PLE, particularly among children with a low genetic risk for schizophrenia. Future studies should consider finding a more robust indicator of the genetic risk of persistent distressing PLE for multi-ancestral populations. Additional research into time-varying neighborhood exposures and their interaction with genetic risk could provide insight into sensitive developmental periods and inform prevention strategies.

## Methods

### Study Population

This study analyzed data from the Adolescent Brain and Cognitive Development (ABCD) Study’s 5.0 release (N = 11,868), collected between September 2016 and January 2022.^25^ The ABCD Study is a nationwide longitudinal investigation of brain and behavioral development across 22 U.S. research sites. Children aged 9 to 10 were recruited at baseline to represent national demographics as closely as possible.^26^ Participating schools (public, private, and charter) were randomly selected within a 50-mile radius of each research site.^26^ All participants and their parents provided written informed consent, with institutional review board approval obtained at each site.

### Persistent distressing psychotic-like experiences

Psychotic-like experiences (PLEs) over the past month were assessed using the Prodromal Questionnaire-Brief Child Version (PQBC).^27, 28^ This screening tool was administered at baseline and three annual follow-ups. For each endorsed PLE item, participants rated their level of distress using a cartoon pictorial 5-point Likert scale, ranging from neutral (1) to extreme distress (5).^27^ Participants were classified as having distressing PLEs if they reported at least one PLE with an associated distress rating ≥ 3.^7^ This threshold was selected to identify clinically meaningful levels of distress that warranted further attention. Among participants with all four years of PLE data (N = 9,912), the persistent distressing PLE group was operationalized as the presence of distressing PLEs at two or more assessment time points.^8^ This definition of persistence was based on previous research on persistent PLEs.^5, 29^

### Genetic Data and Polygenic Risk Score

Genotyping was conducted using the Affymetrix NIDA SmokeScreen Array (733,329 single nucleotide polymorphisms [SNPs]) in 11,666 participants. The genetic dataset was imputed using the TOPMed (Version R2 on GRCh38; https://imputation.biodatacatalyst.nhlbi.nih.gov) reference panel through MiniMac 4, as facilitated by the TOPMed Imputation Server.^30^ The imputed variants, initially represented as fractional dosages, were converted into integer allele counts by applying a best-guess threshold of 0.9. This process yielded a total of 280,985,564 imputed variants, aligned to the GRCh38 genome build. Quality control (QC) was performed in the imputed genetic dataset by removing SNPs with minor allele frequency (MAF) < 1% and Hardy-Weinberg Equilibrium P-value < 1e-6. Population stratification was assessed by conducting the genetic principal component analysis (PCA) using the *plink* --*pca* function in PLINK 1.9 beta (https://www.cog-genomics.org/plink/1.9/). Genetic ancestry was determined using *SNPweights* software^31^ with HapMap 3 reference panels.^32^ Participants were grouped based on whether they had at least 50% genetic similarity to a continental reference panel (African [AFR], East Asian (EAS), or European (EUR) ancestry) or were classified as admixed if no single ancestry exceeded the 50% threshold, indicating a mixture of two or more continental ancestries. Based on participants’ PCs and assigned genetic ancestry, we found the first four PCs adequately distinguished population stratification and were used to adjust for population stratification in the following analyses (Supplementary Fig. 5). Given that the participant recruitment was nested within families (N = 5,678), we calculated the genetic relatedness matrix and used it to account for the dependency between participants using the *plink* -- *make-rel* function.^33^

We used SBayesRC^34^ to calculate a multi-ancestral SCZ-PRS. The summary statistics of schizophrenia from EUR, EAS, and AFR subpopulations were extracted from the latest schizophrenia genome-wide association study (GWAS).^18^ Each individual SCZ-PRS was calculated by incorporating genomic functional annotations and the corresponding UK Biobank (UKB) ancestry-specific LD reference panel with imputed common SNPs (> 7 millions) (https://github.com/zhilizheng/SBayesRC). To construct the multi-ancestral SCZ-PRS, participants with both genetic and PLE phenotype data (N = 9,781) were first randomly split into a training (N = 1,955) and test sample (N = 7,826) in a 20/80 ratio within each ancestry group. We then applied logistic regression to regress persistent distressing PLE on all ancestry-specific SCZ-PRSs in the training sample. Using the coefficients of ancestry-specific SCZ-PRSs as their weights, the multi-ancestral SCZ-PRS was calculated by weighted-summing all ancestry-specific SCZ-PRSs in the test sample (Supplementary Table 5). The final multi-ancestral SCZ-PRS was transformed to the standard normal distribution.

To evaluate the robustness of SBayesRC, we also applied another multi-ancestral PRS approach, PRScsx,^35^ to calculate the SCZ-PRS. The ancestry-specific SCZ-PRS were calculated using the ancestry-specific LD-reference panel using the UKB data before building the multi-ancestral PRS using the same process described above (https://github.com/getian107/PRScsx).

### Neighborhood-level Exposures

Neighborhood-level characteristics were derived from participants’ primary home addresses at baseline. These geospatial location data were geocoded into census tracts and linked to external environmental constructs as part of the ABCD 5.0 release.^25^ We included 29 neighborhood-level characteristics derived from five domains and based on prior literature:^20, 25^ the Area Deprivation Index (ADI),^36, 37^ Child Opportunity Index 2.0 (COI),^38^ Crime,^39^ Environmental Quality,^40, 41^ and the Social Vulnerability Index (SVI).^42^ All characteristics were standardized and coded such that greater values indicated worse conditions. A detailed list of neighborhood-level exposure variables is described in Supplementary Table 6.

### Sociodemographic and Clinical characteristics

At baseline, sociodemographic and clinical characteristics, including age, sex, self-reported race and ethnicity, parental education, income-to-needs ratio, and family history of psychosis, were collected through parent reports and interviews. Race and ethnicity were categorized into non-Hispanic White, non-Hispanic Black, non-Hispanic Asian, non-Hispanic other races, and Hispanic.^43^ Parental education was dichotomized into high (having at least one parent or caregiver who obtained at least a bachelor’s degree) and low (neither parent or caregiver obtained at least a bachelor’s degree). The income-to-needs ratio was calculated by dividing the median value of the income band by the federal poverty line for the respective household size.^44^ A value greater or less than one would denote above or below the poverty threshold. Family history of psychosis in first-degree and second-degree relatives was assessed using the parent-rated Family History Assessment Module Screener.^45^

### Statistical Analysis

To quantify the levels of NE and the relative importance of each neighborhood-level exposure, we used WQS regression^46^ to calculate an NE score for each participant with complete information on neighborhood factors and sociodemographic and clinical characteristics (N = 8,145) using the *gWQS* R package.^47^ Consistent with our previous work,^20^ all neighborhood factors were split into deciles before entering into the WQS regression to calculate the NE score and weights corresponding to the contribution of each exposure to the mixture effect. We adjusted for sex, age, income-to-need ratio, parental education levels, family history of psychosis, and four genetic PCs in WQS regression with constraint to positive unidirectionality.

Using a complete case analysis, 6,449 participants with complete sociodemographic and clinical characteristics in the test sample were included in the final analytic sample. A detailed selection process is provided in Fig. 1. To account for the relatedness of participants in the ABCD cohort by families (N = 5,678) and study sites (N = 22), we used the generalized linear mixed model approach of Chen et al. The model’s formula can have a simplified notation:

$$g\left(E\left(y\right)\right)=X\beta +b_{1i}+b_{2j}$$

$$b_{1i}\sim 𝒩\left(0,V_{1}\right)\;\text{and}\;b_{2i}\sim 𝒩\left(0,V_{2}\right)$$

where *g()* is the link function (*“logit”* here), *E(y)* the expectation value of the outcome, *X* the covariate matrix, *β* the vector of fixed effects, *b*_1*i*_ the random intercept for the *i*^*th*^ participant assuming to follow the normal distribution with mean 0 and covariance proportional to the genetic relatedness matrix *V*_1_, and *b*_2*j*_ the random intercept for the *j*^*th*^ study site assuming to follow the normal distribution with mean 0 and covariance proportional to the block diagonal matrix *V*_2_. The analysis was performed using the *GAMMT* R package (https://github.com/hanchenphd/GMMAT).^48^ We first separately estimated the association of SCZ-PRS calculated by SBayesRC and NE score with persistent distressing PLE, adjusting for sex, age, income-to-need ratio, parental education levels, family history of psychosis, and four genetic PCs. We then conducted a GxE interaction analysis by including SCZ-PRS, NE score, and their interaction term in the model, adjusting for the same covariates. We also assessed the interaction between each individual neighborhood-level exposure and SCZ-PRS to determine which exposures mainly contribute to the GxE interaction. Additive interaction was calculated using the relative excess risk due to interaction (RERI).^49^ Ancestry-stratified analyses were conducted when assessing the association between SCZ-PRS and persistent distressing PLE, as well as the GxE interaction analysis. We report adjusted odds ratios (ORs), 95% confidence intervals (CIs), and two-sided *P*-values (*P*).

Sensitivity analyses included (1) replicating all above analyses using SCZ-PRS calculated by PRScsx, (2) additionally adjusting for self-reported race and ethnicity, and (3) removing participants from the AFR ancestry group in the analysis due to its smallest sample size in GWAS summary statistics used to train multi-ancestral SCZ-PRS (N = 9,824). A two-sided *P* < 0.05 was considered statistically significant. All analyses were performed using R 4.4.0.

## Figures and Tables

**Table 1:** Characteristics of participants in analytic sample from the Adolescent Brain Cognitive Development (ABCD) cohort

Characteristic	N = 6,449
	n (%)
Age, mean (SD)	9.9 (0.6)
Sex	
Female	3,069 (47.6)
Income-to-needs ratio, mean (SD)	4.3 (3.3)
Family history of psychosis	
Yes	517 (8.0)
Parental education	
At least one parent has obtained a bachelor’s degree or greater	4,264 (66.1)
Self-reported race and ethnicity	
Non-Hispanic White	3,718 (57.7)
Non-Hispanic Black	730 (11.3)
Hispanic	1,174 (18.2)
Non-Hispanic Asian	133 (2.1)
Other	694 (10.8)
Genetic ancestry	
EUR	4,985 (77.3)
AFR	706 (10.9)
EAS	168 (2.6)
ADMIX	590 (9.1)
Persistent distressing psychotic-like experiences	
Yes	1,272 (19.7)

## Data Availability

The ABCD study anonymized data are released annually and are publicly available via the NIMH Data Archive (NHA). All data from the Adolescent Brain Cognitive Development (ABCD) Study (https://nda.nih.gov/abcd/request-access) are made available to researchers from universities and other institutions with research inquiries following institutional review board and National Institute of Mental Health Data Archive approval.

## Abbreviations

ADMIX: Admixed ancestry,AFR,African ancestry
EAS: East Asian ancestry
EUR: European ancestry
